# Effect of active and passive warming on preventing hypothermia and shivering during cesarean delivery: a systematic review and meta-analysis of randomized controlled trials

**DOI:** 10.1186/s12884-022-05054-7

**Published:** 2022-09-21

**Authors:** Qing Zhuo, Jia-Bin Xu, Jing Zhang, Bin Ji

**Affiliations:** 1 Fujian Maternity and Child Health Hospital, College of Clinical Medicine for Obstetrics & Gynecology and Pediatrics, Fujian Medical University, Fuzhou, China; 2 Fujian Branch of Shanghai Children’s Medical Center Affiliated to Shanghai Jiaotong University School of Medicine, Shanghai, China; 3 Shanghai Children’s Medical Center, School of Medicine, Shanghai Jiao Tong University, Shanghai, China

**Keywords:** Active warming, Passive warming, Hypothermia, Shivering, Cesarean delivery

## Abstract

**Background:**

Perioperative hypothermia and shivering commonly occur in pregnant women undergoing cesarean section. The warming method is usually used to prevent hypothermia and shivering. However, the effect of active warming (AW) prior to passive warming (PW) on the perioperative outcomes of pregnant women and their offspring remains controversial.

**Methods:**

This study aimed to investigate the effects of AW and PW on maternal and newborn perioperative outcomes during cesarean delivery. According to the Preferred Reporting Items for Systematic Reviews and Meta-Analyses, PubMed, Embase, Scopus, and the Cochrane Library were used to search for randomized controlled trials (RCTs) up to August 7, 2022. The Cochrane risk of bias assessment tool was used to assess articles selected for the systematic review. Continuous data were analyzed using weighted mean differences (WMDs) with 95% confidence intervals (CIs), and categorical data were analyzed by the random-effects model.

**Results:**

A total of 1241 participants from twelve RCTs were selected for the final meta-analysis. AW was associated with a lower risk of maternal hypothermia (RR: 0.77, 95% CI: 0.63–0.93, *P* = 0.007) and shivering (RR: 0.56, 95% CI: 0.37–0.85; *P* = 0.007). AW was associated with high maternal temperature (WMD: 0.27, 95%CI: 0.14 to 0.40, *P* < 0.001). No significant difference was observed between AW and PW in terms of hypothermia (RR: 0.60, 95% CI: 0.24–1.51, *P* = 0.278), temperature (WMD: 0.31, 95% CI: − 0.00 to 0.62; *P* = 0.050), and umbilical vein PH in newborns (WMD: -0.00; 95% CI: − 0.02 to 0.02, *P* = 0.710).

**Conclusions:**

These findings suggested that AW can better prevent maternal hypothermia and shivering than PW. In contrast, no significant effect was observed in newborns. Overall, the quality of the included studies is high due to RCTs, low risk of bias, consistency, and precision. We identified the quality of the overall evidence from the survey to be GRADE I.

**Supplementary Information:**

The online version contains supplementary material available at 10.1186/s12884-022-05054-7.

## Introduction

Hypothermia and shivering are observed in 30 to 60% of parturients during cesarean section with a neuraxial anesthesia [1]. Due to vasodilation of the lower level neuraxial sensory blockade, combined spinal and epidural anesthesia is associated with the reduction in body temperature [1]. Maternal hypothermia causes the progression of maternal shivering and hypothermia in newborn offspring [2]. Shivering is also a common complication in cesarean section (CS). Moreover, perioperative hypothermia and shivering induce side effects such as surgical wound infection, coagulopathy, and an increased blood loss [3, 4].

Both active warming (AW) and passive warming (PW) are often used to prevent perioperative hypothermia and shivering in women with CS. AW interventions are implemented in CS women including forced-air warming, warmed IV fluid, and conduction mattress warming. PW promotes heat retention including cotton blankets or reflective blankets. AW is associated with a reduction in the risk of perioperative hypothermia and shivering in patients undergoing surgery with general or regional anesthesia [5]. The effects of different warming methods have been reported to be heterogeneous in CS populations with neuraxial anesthesia [6, 7]. However, the effect of AW and PW on CS women with CS remains unclear. A good number of studies have already compared the effects of AW with PW for pregnant women undergoing cesarean delivery. The effect between AW and PW on hypothermia and shivering remains controversial due to bias factors including anesthetics, temperature site, amniotic fluid volume, and warming duration [6, 7]. Therefore, a systematic review and meta-analysis of randomized controlled trials (RCTs) was conducted to investigate the effects of AW and PW on preventing hypothermia and shivering in pregnant women undergoing cesarean delivery.

## Methods

### Data sources, search strategy, and selection criteria

The systematic review and meta-analysis were conducted according to the Preferred Reporting Items for Systematic Reviews and Meta-Analyses guidelines [8]. Eligible studies were published in English, RCTs, comparisons of the effects of AW and PW on maternal and newborn perioperative outcomes, and no restrictions on publication status. PubMed, Embase, Scopus, and the Cochrane library were searched until August 7, 2022. The following keywords and medical terms were employed in electronic literature searches: (“cesarean section” OR cesarean OR caesarean) AND (epidural OR spinal OR regional OR local) AND (anesthesia OR aneasthesia) AND (“warming techniques” OR “heating” OR “carbon fiber” OR “forced air” OR “circulating water garment*” OR vital heat OR vital heat OR “bair hugger*” OR “hot dog” OR hotdog OR “bair paw*” OR heat OR heated OR heating OR normothermia OR normothermic OR warm OR warming OR warmed OR warmth OR hot OR rewarming). The retrieved studies were manually reviewed as potential new eligible studies.

Two authors conducted the literature search and study selection in accordance with a standard flow. Conflicts were resolved by discussion with each other until a consensus was obtained. The inclusion criteria of this study were listed as follows: (1) Participants: all of participants were pregnant women over 18 years old undergoing cesarean delivery, regardless of anesthesia approach. (2) Intervention: AW. (3) Control: PW. (4) Outcomes: The study reported perioperative outcomes including maternal (hypothermia, shivering, temperature) and newborn (hypothermia, temperature, and umbilical vein PH in newborns) outcomes. (5) Study design: RCT. The exclusion criteria were defined below: (1) participants: non-human. (2) not designed control group. (3) not reported the major-outcomes including hypothermia and shivering. (4) non-RCTs. (5) publications reported using a non-English language.

### Data collection and quality assessment

The data were abstracted by 2 authors respectively, and disagreement was settled by a group discussion. The items collected included the first author’s name, publication year, country, sample size, mean age, anesthesia, intervention, control, temperature site, cutoff of hypothermia, and outcomes. The quality of the included studies was assessed using the Cochrane Collaboration Tool [9]. Studies were assessed according to random sequence generation, allocation concealment, double-blind to participants and outcome assessor, outcome report, and sample size calculation. The quality of studies was ranked as a low, high, or unclear risk of bias.

### Statistical analysis

The results of individual RCTs were assigned as dichotomous and continuous data. Relative risk (RR) and weighted mean difference (WMD) with associated 95% confidential intervals (CIs) were calculated from each trial before data pooling. The total effect of AW versus PW was analyzed using the random-effects model [10, 11]. Heterogeneity was assessed using the I-square and Q statistics, and *P* < 0.10 was identified as significant heterogeneity [12, 13]. The robustness of the pooled conclusion was evaluated using the sensitivity analysis excluding individual trials [14]. Subgroup analyses were conducted on the basis of anesthesia, temperature site, and study quality. The difference between both subgroups were estimated using the interaction *P* test [15]. Funnel plots and Egger’s and Begg’s test results were used to assess any potential publication bias [16, 17]. Given that the significant publication bias was detected, the results were adjusted using the trim and fill method [18]. All *P* values were two-sided with a significance level of 0.05. The Stata software (version 10.0, Stata Corporation, College Station, Texas) was used for the analysis in this study.

## Results

### Search of the published literature

**Fig. 1:**
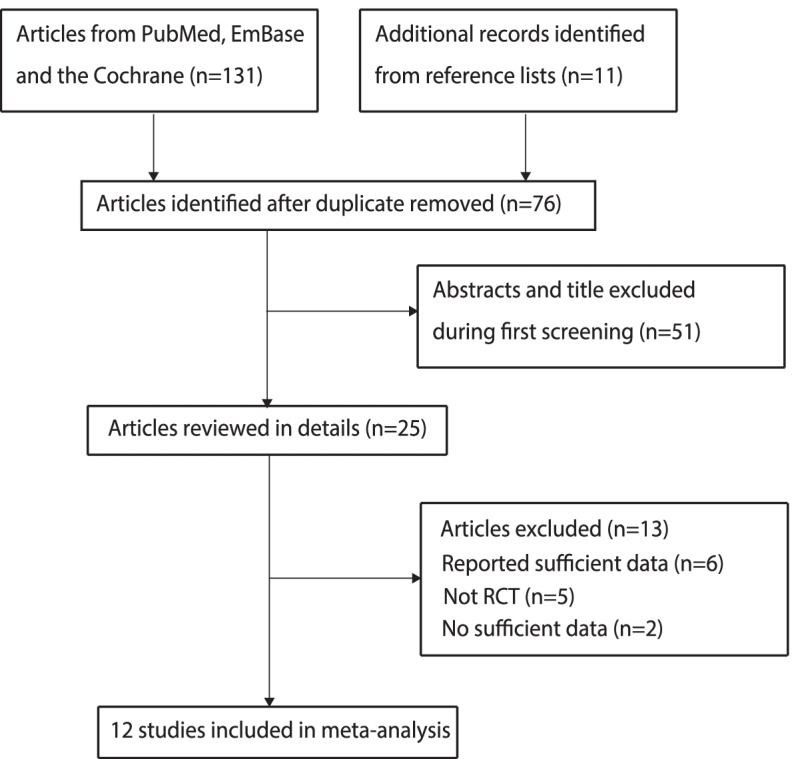
Flow diagram showing the study selection process

The initial electronic search produced 131 records, and 55 studies were excluded as duplicates. An additional 51 studies were eliminated as irrelevant afte reviewing their titles and abstracts. In addition, 12 studies were excluded for the following reasons: the study reported the same population (*n* = 6), was not an RCT (*n* = 5), and did not have sufficient data (*n* = 2). Reviewing the references of the remaining studies yielded 12 records without new studies identified by hand-search. Finally, 12 RCTs met the inclusion criteria and were included in our meta-analysis (Fig. 1) [19–30].

### Characteristics of the included studies

**Fig. 2:**
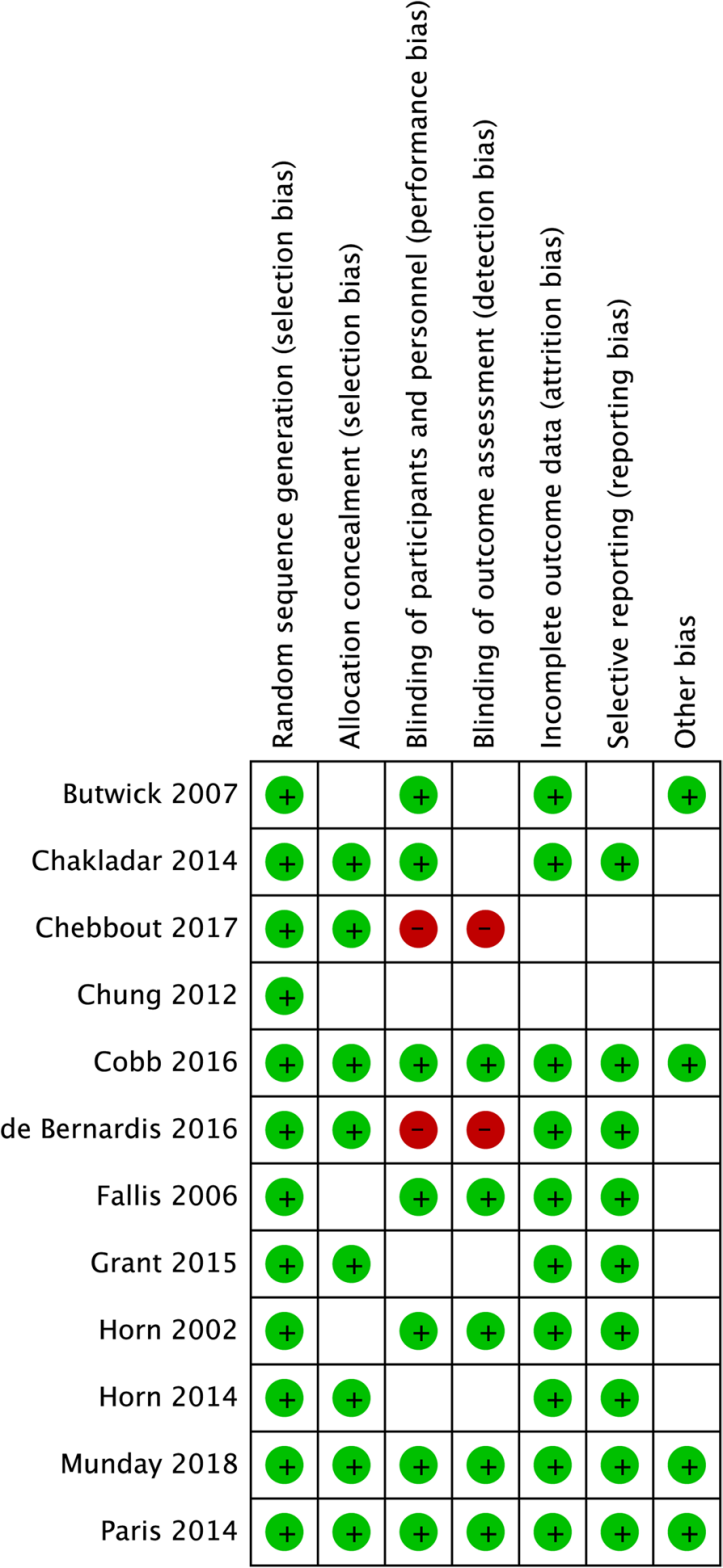
Risk of bias summary on each included study

Table 1 summarizes in Table 1 about the baseline characteristics of the included studies. Overall, 12 RCTs involved a total of 1241 pregnant women undergoing cesarean delivery who were selected for final quantitative analyses. These studies were published between 2002 and 2018, and 30–484 individuals were included in each trial. The mean age of the pregnant women ranged from 28.8–34.0 years. Five of the included trials were conducted in the US, 2 trials were performed in the UK, and the remaining 5 studies were carried out in Canada, Korea, Germany, Brazil, and Australia, respectively. According to Review manager version 5.4.1, the quality of included studies was high (Fig. 2).

**Table 1:** Baseline characteristic of studies included in the systematic review and meta-analysis

Study	Publication year	Country	Sample size	Mean age (years)	Anesthesia	Intervention	Control	Temperature site	Hypothermia Cutoff
Horn [19]	2002	US	30	32.0	Epidural	FAW on upper body with 15- minute pre-operation warming on full body, i.v. warm fluids	Cotton blanket, i.v. warm fluids	Tympanic and skin	NA
Fallis [20]	2006	Canada	62	30.0	Spinal	FAW on upper body, i.v. warm fluids	Cotton blanket, i.v. warm fluids	Oral	36.5
Butwick [21]	2007	US	30	34.0	Spinal	FAW on lower extremities	FAW blanket turned off	Oral	35.5
Chung [22]	2012	Korea	30	32.2	Spinal	FAW on upper body with, i.v. warm fluids 15-minute pre-op warming	FAW blanket turned off	Tympanic and skin	NA
Horn [23]	2014	Germany	40	31.0	Spinal	FAW on upper body	Warm blankets	Oral and skin	36.0
Paris [24]	2014	US	226	31.7	Spinal	Underbody conductive heat mat, i.v. warm fluids with pre-op warming	Warm blankets	Oral and bladder	36.0
Chakladar [25]	2014	UK	116	34.0	Spinal, epidural, and general	Underbody conductive heat mat, i.v. warm fluids if> 500 ml administered	Cotton sheet, warm fluids if> 500 ml administered	Tympanic	36.0
Grant [26]	2015	US	484	NA	Spinal, combined spinal-epidural, general	Underbody conductive heat mat, warm blanket, reflective cap, i.v.warm irrigation fluids	Warm blanket, reflective cap, i.v.warm irrigation fluids	Oral and bladder	36.0
Cobb [27]	2016	US	46	31.5	Spinal	FAW on lower extremities, i.v. warm fluids	Cotton blankets	Temporal artery and bladder	36.0
de Bernardis [28]	2016	Brazil	40	28.8	Spinal	FAW from a thermal gown	Regular blankets	Tympanic	36.0
Chebbout [29]	2017	UK	87	31.8	Spinal	FAW and i.v. warm fluids	i.v. warm fluids	Oral	36.5
Munday [30]	2018	Australia	50	33.5	Spinal, Epidural	FAW and i.v. warm fluids	i.v. warm fluids	Tympanic and bladder	36.0

*FAW *forced air warming

### Maternal outcomes

**Fig. 3:**
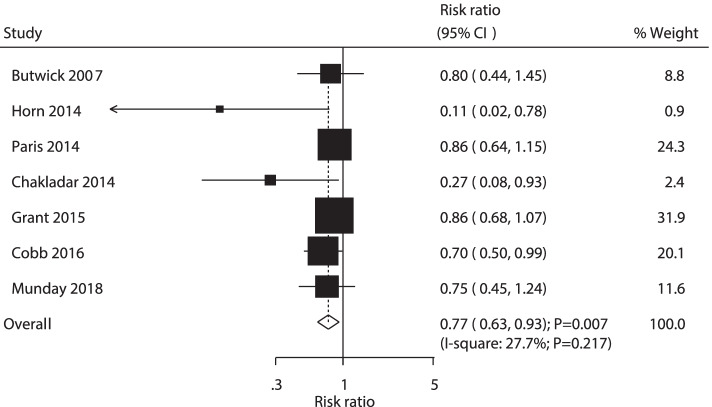
Forest plot showing the comparison between active and passive warming on the risk of hypothermia in mothers

A total of 7 RCTs reported the effect of AW versus PW on the risk of maternal hypothermia. We found that AW was associated with a reduced risk of hypothermia (RR: 0.77; 95% CI: 0.63–0.93; *P* = 0.007; Fig. 3). The heterogeneity was not significant in the included trials (*P* = 0.217). The pooled conclusion for the risk of maternal hypothermia was robust and not altered with sequential exclusion of individual trials. Significant differences were detected by subgroup analyses between AW and PW in maternal hypothermia, divided by temperature sites and study quality (Table 2). Moreover, the publication bias of maternal hypothermia (*P* value for Egger: 0.008, *P* value of Begg: 0.035, Supplemental Fig. 1) and the conclusion by the trim and fill method were unaltered (Supplemental Fig. 2).

**Table 2:** Subgroup analyses for investigated outcomes

Outcomes	Factor	Groups	RR or WMD and 95%CI	*P* value	Heterogeneity (%)	*P* value for heterogeneity	*P* value between subgroups
Hypothermia in maternal	Anesthesia	Spinal	0.75 (0.55–1.02)	0.065	39.1	0.177	1.000
		Other	0.75 (0.51–1.08)	0.124	41.5	0.181	
	Temperature site	Oral	0.82 (0.65–1.05)	0.114	31.1	0.226	0.287
		Other	0.66 (0.46–0.96)	0.029	28.8	0.245	
	Study quality	High	0.71 (0.54–0.94)	0.017	38.9	0.147	0.732
		Low	0.86 (0.68–1.07)	0.170	–	–	
Shivering in maternal	Anesthesia	Spinal	0.58 (0.38–0.88)	0.011	0.0	0.434	0.831
		Other	0.52 (0.18–1.52)	0.233	63.5	0.065	
	Temperature site	Oral	0.79 (0.44–1.42)	0.432	0.0	0.431	0.219
		Other	0.47 (0.28–0.81)	0.006	30.4	0.207	
	Study quality	High	0.56 (0.32–0.97)	0.037	29.0	0.228	1.000
		Low	0.52 (0.23–1.19)	0.122	46.0	0.157	
Temperature in maternal	Anesthesia	Spinal	0.18 (0.06 to 0.31)	0.004	52.6	0.077	0.059
		Other	0.42 (0.10 to 0.75)	0.011	91.0	< 0.001	
	Temperature site	Oral	0.16 (0.07 to 0.24)	< 0.001	42.2	0.140	0.003
		Other	0.52 (0.06 to 0.97)	0.025	89.3	< 0.001	
	Study quality	High	0.39 (0.11 to 0.66)	0.006	86.5	< 0.001	0.123
		Low	0.16 (0.09 to 0.23)	< 0.001	4.2	0.352	
Temperature in newborn	Anesthesia	Spinal	0.22 (−0.19 to 0.62)	0.295	95.3	< 0.001	0.006
		Other	0.53 (−0.16 to 1.21)	0.132	90.1	0.001	
	Temperature site	Oral	0.22 (−0.19 to 0.62)	0.295	95.3	< 0.001	0.006
		Other	0.53 (−0.16 to 1.21)	0.132	90.1	0.001	
	Study quality	High	0.53 (0.07 to 1.00)	0.025	95.6	< 0.001	0.001
		Low	−0.10 (−0.29 to 0.10)	0.325	50.0	0.157	
Umbilical vein PH	Anesthesia	Spinal	−0.01 (−0.02 to − 0.00)	0.009	26.2	0.254	< 0.001
		Other	0.08 (0.03 to 0.13)	0.002	–	–	
	Temperature site	Oral	−0.01 (−0.04 to 0.02)	0.511	52.5	0.147	0.586
		Other	0.01 (−0.03 to 0.05)	0.777	86.0	0.001	
	Study quality	High	0.00 (−0.02 to 0.03)	0.784	79.7	0.002	0.167
		Low	−0.03 (−0.06 to − 0.00)	0.040	–	–	

**Fig. 4:**
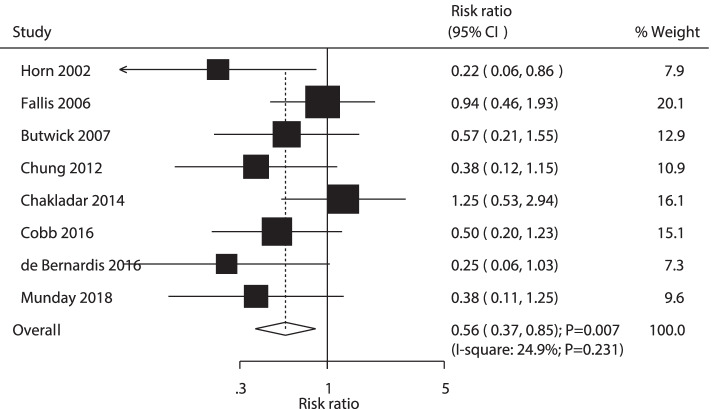
Forest plot comparing the effects of active and passive warming on maternal shivering

A total of 8 RCTs reported the effect of AW versus PW on the risk of maternal shivering. The summary RR indicated that AW versus PW was associated with a lower risk of maternal shivering (RR: 0.56; 95% CI: 0.37–0.85; *P* = 0.007; Fig. 4). The heterogeneity of the included trials was not significant (*P* = 0.231). No significant difference was found using sensitivity analysis based on excluding one-by-one exclusion. Subgroup analyses indicated that AW versus PW was associated with a lower risk of maternal shivering with spinal anesthesia, assessed by temperature sites or study quality (Table 2). There was significant publication bias for maternal shivering (*P* value for Egger: 0.003; *P* value for Begg: 0.019; Supplemental Fig. 3), and the conclusion was stable under adjusted potential publication bias (Supplemental Fig. 4).

**Fig. 5:**
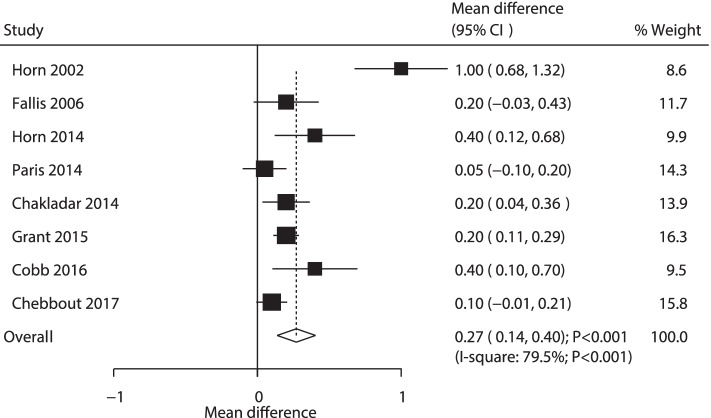
Forest plot comparing the effects of active and passive warming on maternal temperature

A total of 8 RCTs reported the effect of AW versus PW on maternal temperature. It was found that AW was related to high temperature as compared with passive warming (WMD: 0.27; 95% CI: 0.14 to 0.40; *P* < 0.001; Fig. 5). Moreover, substantial heterogeneity was observed among the included studies (*P* < 0.001). The conclusion was stable and not changed by excluding one-by-one exclusion. The results of subgroup analyses were consistent with the overall analysis using all subgroups (Table 2). Although the Begg’s test indicated no significant publication bias (*P* = 0.108), Egger’s test suggested potential publication bias of maternal temperature (*P* = 0.083). The conclusions remained unchanged under adjustment for publication bias by the trim and fill method.

### Newborn outcomes

**Fig. 6:**
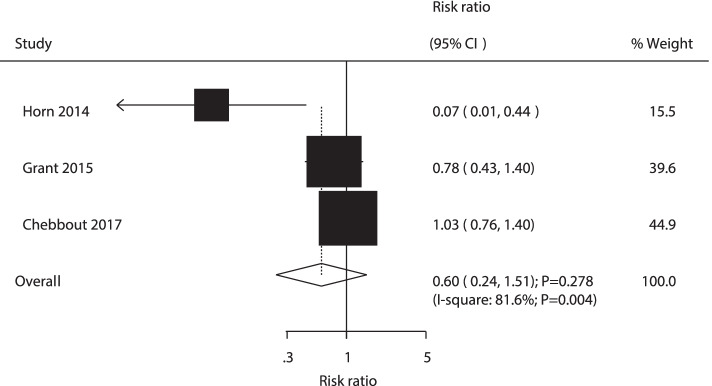
Forest plot comparing the effects of active and passive warming on neonatal hypothermia

A total of 3 RCTs reported the effect of AW versus PW on the risk of neonatal hypothermia. There was no significant difference between active and passive warming in the risk of neonatal hypothermia (RR: 0.60; 95% CI: 0.24–1.51; *P* = 0.278; Fig. 6). The heterogeneity was significant among the included trials (*P* = 0.004).

**Fig. 7:**
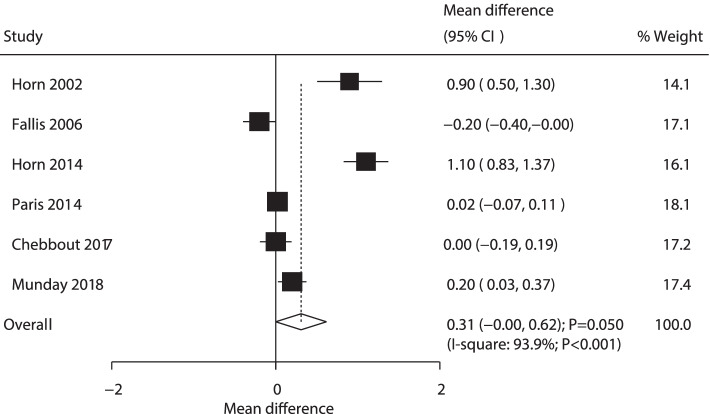
Forest plot comparing the effects of active and passive warming on neonatal temperature

A total of 6 RCTs published the effect of AW versus PW on neonatal temperature. AW did not yield a significant effect on neonatal temperature compared with PW (WMD: 0.31; 95% CI: − 0.00 to 0.62; *P* = 0.050; Fig. 7). The heterogeneity was detected to be significant across the included studies (*P* < 0.001). Sensitivity analysis indicated that active warming was associated with high temperature, given excluding the trial using 36.5 °C as a cutoff value of hypothermia (Supplemental Fig. 1) that conducted by Fallis et al. [20]. Subgroup analysis found that AW rather than PW was associated with high temperature in newborns when pooled studies were of high quality (Table 2). No significant publication bias was detected in neonatal temperature (*P* value for Egger: 0.179, *P* value for Begg: 0.452, Supplemental Fig. 2).

**Fig. 8:**
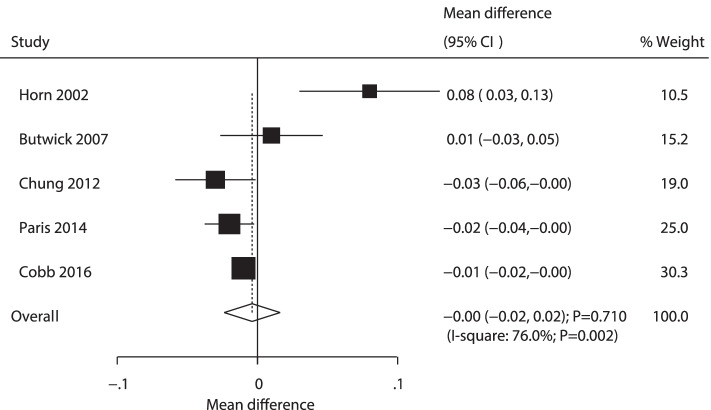
Forest plot comparing the effects of active and passive warming on umbilical vein pH in newborns

A total of 5 RCTs reported the effect of AW versus PW on umbilical vein PH in newborns. No significant difference was observed between active and passive warming on umbilical vein PH in neonates (WMD: -0.00; 95% CI: − 0.02 to 0.02; *P* = 0.710; Fig. 8). The heterogeneity was found to be significant among the included trials (*P* = 0.002). Sensitivity analysis showed that active warming might be more closely associated with lower umbilical vein PH in newborns than passive warming (Supplemental Fig. 1). Subgroup analyses indicated a reduction in umbilical vein PH in PW newborns was detected in participants receiving spinal anesthesia, and in low-quality studies. In addition, participants receiving other anesthesia with AW were associated with high umbilical vein PH in newborns (Table 2). No significant publication bias for umbilical vein PH in newborns was observed (*P* value for Egger: 0.547; *P* value for Begg: 0.462; Supplemental Fig. 2).

## Discussion

In this study, we found that AW decreased the incidence of maternal hypothermia and shivering compared with PW. In contrast, there was no difference in neonatal temperature or umbilical vein PH between AW and PW. Moreover, the treatment outcomes of AW and PW might be affected by anesthesia, temperature site, and study quality. Thus, AW occurs prior to PW in preventing perioperative hypothermia during CS.

Under neuraxial anesthesia, AW reduces intraoperative hypothermia more effectively than PW [1]. Consistent with a previous review [1], our study confirmed that AW has a prior advantage of preventing perioperative hypothermia and shivering in women with CS regardless of anesthesia modes. A previous systematic review including 25 studies illustrated that AW is superior to PW in preventing hypothermia in patients with a neuraxial anesthesia. In addition, AW is associated with high temperature rather than PW. A great deal of bias resulted from patients undergoing various surgeries. Considering the confusing factors of surgery, our study focused on pregnant women undergoing cesarean delivery.

Our findings demonstrated that most of the included trials reported that AW was associated with high maternal temperature. However, 3 of the included trials did not show significant differences between AW and PW. A good explanation is that redistribution of intravascular volume from the core to the peripheral compartment below the level of sympathectomy, which contributes to more radiant loss of body heat [3, 31, 32]. Forced-air warming combined with the injection of warmed fluids could minimize core temperature loss. Compared with forced-air warming of the patient from outside, injection of warmed fluids could decrease body temperature much mor slowly body temperature on account of a redistribution hypothermia. The intensity and incidence of shivering are significantly correlated with hypothermia [20, 24, 29].

This study illustrated that there were no significant differences between AW and PW in terms of hypothermia, temperature, or umbilical vein pH in newborns. This finding can be explained by the small sample size of the included trials. In addition, the temperature of newborn infants might be determined by the duration spent to handling babies after delivery and then skin-to-skin care [33]. Factors involved in the incidence of neonatal hypothermia after delivery include prematurity, low birth weight, low Apgar score, and antenatal steroid administration [34].

Subgroup analyses indicated that temperature site exert an effect on maternal and newborn temperature, anesthesia type had an effect on neonatal temperature and umbilical vein pH, and study quality had an effect on neonatal temperature. There are several reasons for these results: (1) Various temperature sites accounted for the differences between AW and PW in maternal and neonatal temperature. (2) Anesthesia type resulted in uncontrolled biases in the conditional variation of pregnant women. (3) Varied study quality of individual trials.

Several limitations should be considered in this meta-analysis. First, the confusion bias items from the role of pharmacological warming in AW and PW. Second, the lack of reported neonatal characteristics unavailable in most of the included trials could affect newborn outcomes. Third, subgroup analyses were insufficient to deal with substantial heterogeneity lying in the included trials. Finally, publication bias was inevitable for publication bias.

In conclusion, the findings of this study indicated that AW is superior to PW in preventing maternal hypothermia and shivering. Further large-scale RCTs should be conducted to investigate the effect of AW and PW on newborn outcomes during cesarean delivery. Overall, the quality of the included studies is high due to RCTs, low risk of bias, consistency, and precision. We identified the quality of the overall evidence from the study to be GRADE I.

## Supplementary Information

**Additional file 1:** **Supplemental Figure 1.** Funnel plot comparing the publication bias of maternal hypothermia.

**Additional file 2:** **Supplemental Figure 2.** Filled funnel plot t comparing the publication bias of maternal hypothermia.

**Additional file 3:** **Supplemental Figure 3.** Funnel plot comparing publication bias for maternal shivering.

**Additional file 4:** **Supplemental Figure 4.** Funnel plot comparing adjusted publication bias for maternal shivering.

## Data Availability

The datasets used in this manuscript is available from the corresponding author on a reasonable request.
