# Supplementary figures and images for: Effect of active and passive warming on preventing hypothermia and shivering during cesarean delivery: a systematic review and meta-analysis of randomized controlled trials
Source: BMC Pregnancy Childbirth. 2022 Sep 21;22:720. doi: 10.1186/s12884-022-05054-7 (PMC9494806; doi:10.1186/s12884-022-05054-7)

Funnel plot with pseudo 95% confidence limits

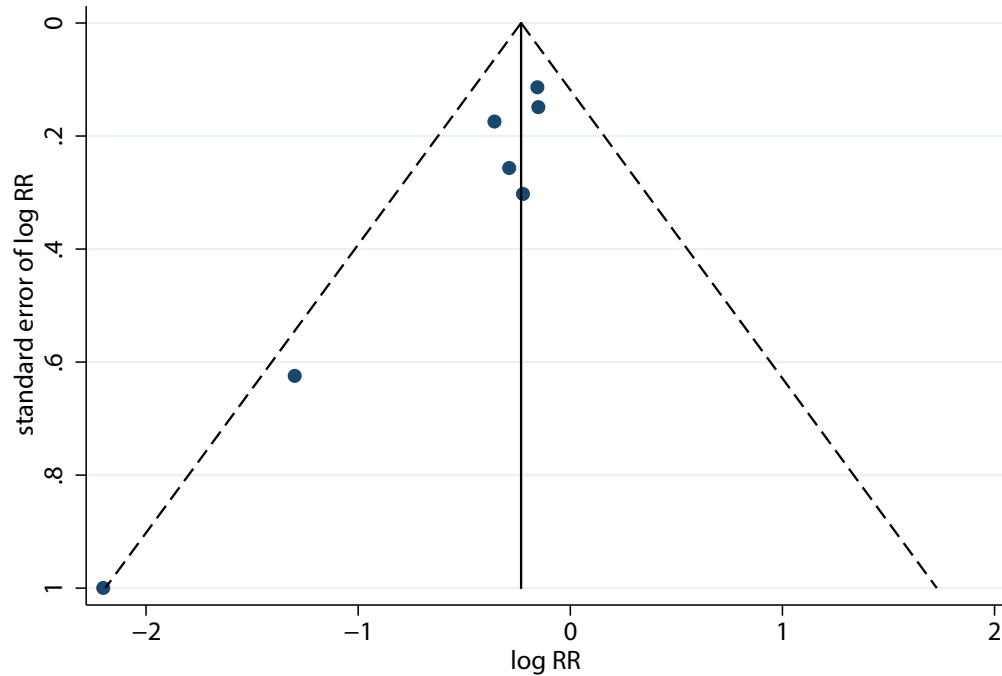

P value for Egger: 0.008

P value for Begg: 0.035

Supplement: Supplementary file 1 — Additional file 1: Supplemental Figure 1. Funnel plot comparing the publication bias of maternal hypothermia. [file 12884_2022_5054_MOESM1_ESM.pdf]

Filled funnel plot with pseudo 95% confidence limits

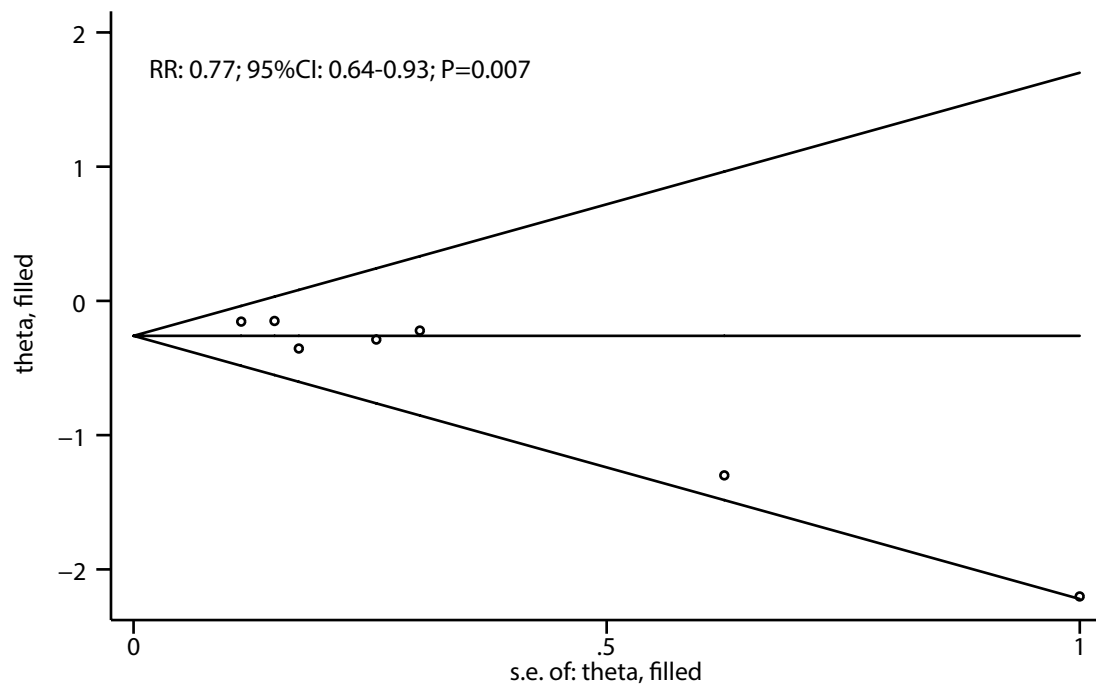

Supplement: Supplementary file 2 — Additional file 2: Supplemental Figure 2. Filled funnel plot t comparing the publication bias of maternal hypothermia. [file 12884_2022_5054_MOESM2_ESM.pdf]

Funnel plot with pseudo 95% confidence limits

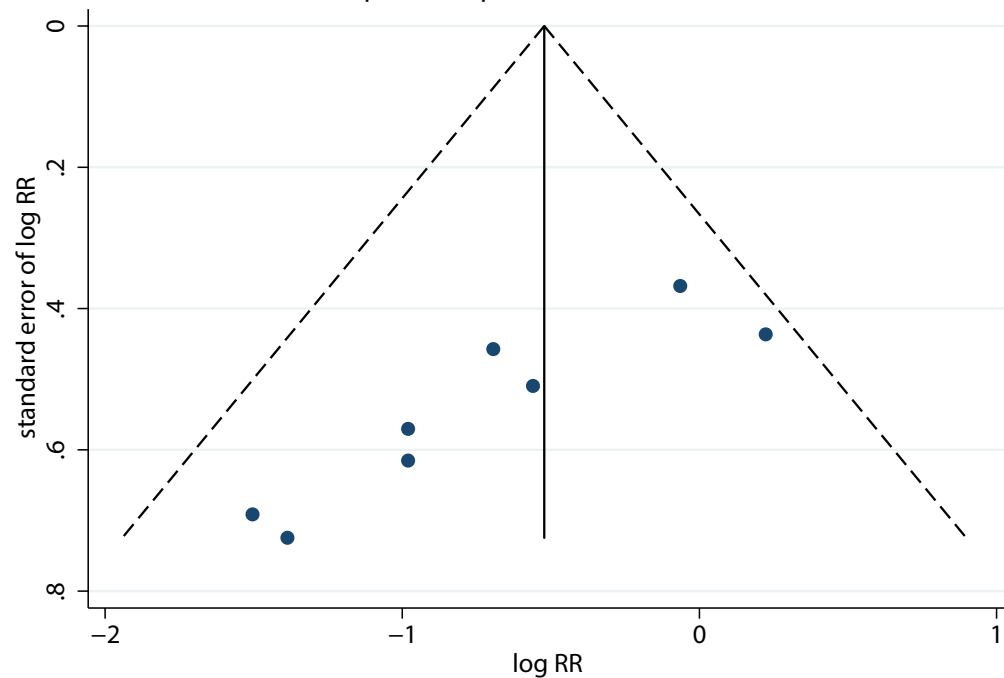

P value for Egger: 0.003

P value for Begg: 0.019

Supplement: Supplementary file 3 — Additional file 3: Supplemental Figure 3. Funnel plot comparing publication bias for maternal shivering. [file 12884_2022_5054_MOESM3_ESM.pdf]

Filled funnel plot with pseudo 95% confidence limits

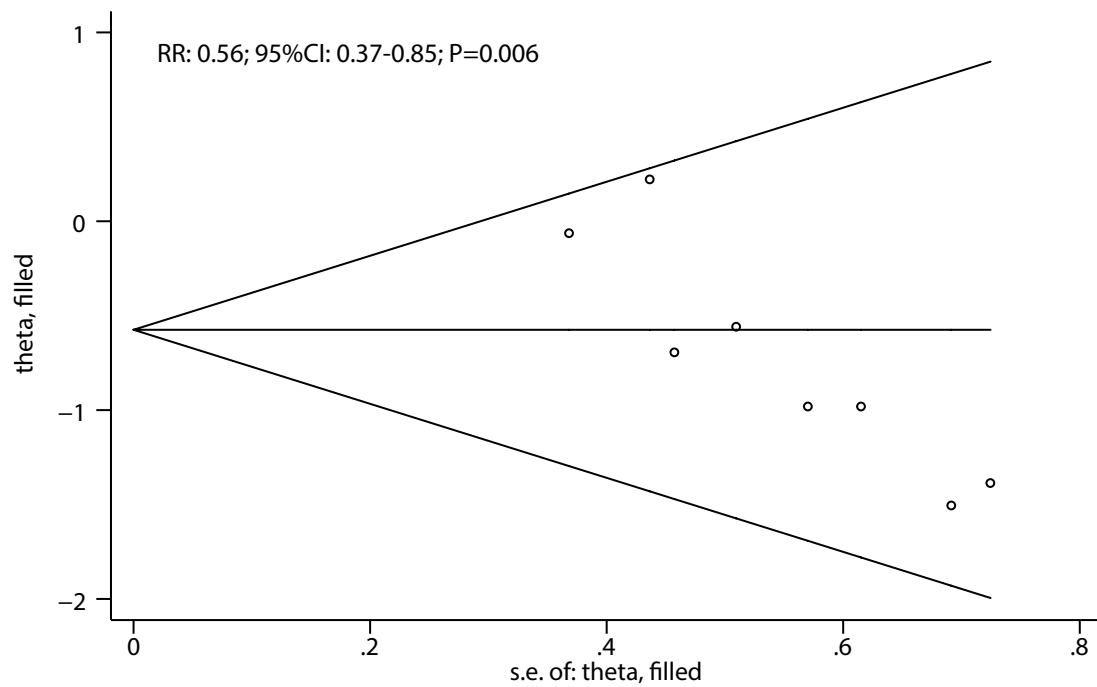

Supplement: Supplementary file 4 — Additional file 4: Supplemental Figure 4. Funnel plot comparing adjusted publication bias for maternal shivering. [file 12884_2022_5054_MOESM4_ESM.pdf]
